# Analysis of Pembrolizumab in Human Plasma by LC-MS/HRMS. Method Validation and Comparison with Elisa

**DOI:** 10.3390/biomedicines9060621

**Published:** 2021-05-30

**Authors:** Aurélien Millet, Nihel Khoudour, Jérôme Guitton, Dorothée Lebert, François Goldwasser, Benoit Blanchet, Christelle Machon

**Affiliations:** 1 Biochemistry and Pharmacology-Toxicology Laboratory, Lyon-Sud Hospital, Hospices Civils de Lyon, F-69495 Pierre Bénite, France; aurelien.millet@chu-lyon.fr (A.M.); christelle.machon@univ-lyon1.fr (C.M.); 2 Department of Pharmacokinetics and Pharmacochemistry, Cochin Hospital, AP-HP, CARPEM, 75014 Paris, France; nihel.khoudour@aphp.fr (N.K.); benoit.blanchet@aphp.fr (B.B.); 3 Toxicology Laboratory, Faculty of Pharmacy ISPBL, University of Lyon 1, F-69373 Lyon, France; 4 Promise Proteomics, 7 Parvis Louis Néel, F-38040 Grenoble, France; dorothee.lebert@promise-proteomics.com; 5 Department of Medical Oncology, Cochin Hospital, AP-HP, CARPEM, 75014 Paris, France; francois.goldwasser@aphp.fr; 6 Analytical Chemistry Laboratory, Faculty of Pharmacy ISPBL, University Lyon 1, F-69373 Lyon, France

**Keywords:** pembrolizumab, mass spectrometry, ELISA, therapeutic drug monitoring

## Abstract

Pembrolizumab is a humanized immunoglobulin G4-kappa anti-PD1 antibody used in the treatment of different solid tumors or haematological malignancies. A liquid chromatography coupled with a high resolution mass spectrometry (orbitrap technology) method was fully developed, optimized, and validated for quantitative analysis of pembrolizumab in human plasma. A mass spectrometry assay was used for the first time a full-length stable isotope-labelled pembrolizumab-like (Arginine ^13^C_6_-^15^N_4_ and Lysine ^13^C_6_-^15^N_2_) as an internal standard; the sample preparation was based on albumin depletion and trypsin digestion and, finally, one surrogate peptide was quantified in positive mode. The assay showed good linearity over the range of 1–100 μg/mL, a limit of quantification at 1 μg/mL, excellent accuracy from 4.4% to 5.1%, and also a between-day precision below 20% at the limit of quantification. In parallel, an in-house ELISA was developed with a linearity range from 2.5 to 50 µg/mL. Then, results were obtained from 70 plasma samples of cancer patients that were treated with pembrolizumab and quantified with both methods were compared using the Passing-Bablok regression analysis and Bland-Altman plotting. The LC-MS/HRMS method is easy to implement in the laboratory for use in the context of PK/PD studies, clinical trials, or therapeutic drug monitoring.

## 1. Introduction

Cancer treatments are based on different strategies generally combining several approaches such as chemotherapy, surgery, and radiotherapy. Immunotherapy with immune checkpoint inhibitors (ICI) is a new class of drugs, which emerged ten years ago with Ipilimumab, an anti-CTLA-4 monoclonal antibody (mAb) [1]. The ICIs essentially take the ‘brakes’ off the immune system, which helps it recognize and attack cancer cells. More recently, PD-1 blocking therapies such as nivolumab and pembrolizumab (PBZ) showed clinical benefits in different cancer treatments [2,3]. PBZ is a humanized IgG4 anti-PD1 used in the treatment of various solid tumors such as lung cancer, melanoma, head, urothelial carcinoma, neck squamous cancer, and some haematological malignancies [4]. PBZ presents a long-term half-life of 27.3 days [5], and the dose approved is not based on body weight but corresponds to a flat dose of 200 or 400 mg administered every 3 or even 6 weeks, respectively [6,7].

Relationships between plasma exposure and efficacy or toxicity have not yet been clearly established for PBZ. However, Phase I studies showed that the receptors were saturated at 1 mg/kg of PBZ every 3 weeks [8], which suggests that therapeutic drug monitoring (TDM) of PBZ could be an interesting strategy to determine the minimum effective concentration to individually modulate the administrated doses or to delay the next administration [9,10]. Recently, a retrospective single-center study conducted in patients with relapsed/refractory Hodgkin lymphoma showed that a low dose of PBZ (100 mg every 3 weeks) provided better results in terms of response and adverse effects than those reported within phase I and II studies performed with high-doses (200 mg or 2 mg/kg every 3 weeks) [11]. This individualization of dosing could allow for reducing the hospital costs without loss of clinical benefit for cancer patients treated with PBZ.

Two ELISA assays have recently been applied to assay PBZ in human serum from cancer patients [12,13]. For the last few years, many studies have reported interests of mass spectrometry methods to determine concentrations of mAbs in human serum [14,15]. To our best knowledge, only one study described a LC-MS/MS assay for the determination of PBZ in human plasma samples [16]. This method used two internal standards: one added in the sample, and the other was post-column infused during the chromatographic analysis.

For a significant period, the ELISA methods have been the reference methods for the determination of proteins. However, in recent years LC-MS has become a powerful alternative for the accurate and reproducible quantification of proteins [17]. In the present work, we described a quick, easy implementation in routine practice and reliable quantification method of PBZ in human plasma with liquid chromatography coupled with a high-resolution-mass-spectrometry (LC-MS/HRMS). This assay was based on a simple albumin depletion protocol of sample preparation and the use of stable-isotope-labelled pembrolizumab-like (SIL-PBZ-like) as an internal standard. The present LC-MS/HRMS was cross validated with an in-house ELISA assay using plasma from 70 cancer patients treated with PBZ.

## 2. Materials and Methods

### 2.1. LC-MS/HRMS

#### 2.1.1. Chemicals and Reagents

PBZ (Keytruda^®^, 25 mg/mL, MSD, Kenilworth, NJ, USA) was kindly provided by the institutional pharmacy. Stable isotope-labeled Pembrolizumab-like (SIL-PBZ-like) was purchased from Promise Advanced Proteomics (Grenoble, France). SIL-PBZ-like is an IgG4-kappa containing three proteotypic peptides of PBZ, which has a purity > 95% and a labelling of arginine and lysine >99%. Stock solutions of PBZ and SIL-PBZ-like were prepared in PBS at 1 g/L and at 100 mg/L, respectively, and stored at +4 °C. Appropriated standard solutions were made daily by further dilution of stock solutions with PBS.

Mobile phases were prepared using ultrapure water obtained from a Milli-Q Plus^®^ system (Millipore, Molsheim, France), ULC/MS grade acetonitrile and methanol from Biosolve (Dieuze, France) and formic acid (FA) from Fisher Chemicals (Illkirch, France). The PBS buffer (pH 7.4, 10X) was from Gibco (Thermo Fisher, Waltham, MA, USA). Trypsin Gold, Mass Spectrometry Grade was purchased from Promega (Madison, WI, USA).

Extraction solvents were prepared using propan-2-ol and trichloroacetic acid 20% for analysis from Carlo Erba Reagents (Val-de-Reuil, France). Ammonium bicarbonate for mass spectrometry was purchased from Sigma-Aldrich (Saint-Quentin-Fallavier, France). Drug-free human serum was provided by the regional blood service (EFS Rhône-Alpes, France). Low adsorption polypropylene microtubes were purchased from Dutsher (Brumath, France).

#### 2.1.2. LC-MS/HRMS Analysis

##### Liquid Chromatography Conditions

Ultimate 3000 chain (Thermo Scientific, Bremen, Germany) was used as an ultra-high pressure chromatographic system. This system was composed of two ternary pumps (left and right), an autosampler maintained at 10°C, and a column oven set at 50 °C. The chromatographic separation of peptides was performed using a Biozen Peptide-PS-C18 (100 × 2.1 mm, 1.6 µm) (Phenomenex, Torrance, CA, USA) preceded by an on-line solid-phase-extraction (SPE) (Strata^TM^-X; 20 × 200 mm, 25 µm, Phenomenex, Torrance, CA, USA). The mobile phase was composed of water with 0.1% of formic acid (A) and acetonitrile with 0.1% of formic acid (B).

During the loading step, the left pump deposited the sample on the on-line SPE and delivered 90% of A at 150 µL/min, while the right pump delivered 85% of A at 150 µL/min. After 1 min, a switch of the valve allowed the elution of peptides from the SPE by the right pump during 30 s from 15% to 50% of B). Then, the valve switched again and the right pump realized the chromatographic separation of peptides under following conditions: 1.5–5 (50–64% B), 5–5.1 (64–90% B), 5.1–6.6 (90% B), 6.6–6.7 (90–15% B), 6.7–11 min (re-equilibration at 15% of B).

##### Mass Spectrometry Configuration

Detection was performed on a Q-Exactive Plus Orbitrap mass spectrometer (Thermo Scientific, Bremen, Germany) coupled with a heated electrospray ionization source (HESI-II). Positive ionization of peptides was carried out under the following conditions: spray voltage at 4 kV, capillary temperature at 320 °C, sheath gas flow rate 25 (arbitrary unit, a.u.), auxiliary gas flow rate 10 a.u., no sweep gas, S-lens RF level 60 V, and auxiliary gas heater temperature at 300 °C. Analyses were performed in parallel reaction monitoring (PRM) mode, with a resolution of 70,000, and an MS1 isolation window of 0.4 Da. The AGC Target value was set at 1,000,000, whereas the maximum injection time was 256 ms. Precursors ions were fragmented at 23 eV of collisional energy in a higher-energy collisional dissociation (HCD) cell, with the first mass of daughter ions fixed at 300 Da. Data collection and process were performed in Xcalibur 2.1 software.

#### 2.1.3. Selection of Peptides for Quantification

The selection of surrogate peptides for a bottom-up approach was performed using tryptic proteolysis of PBZ in silico with Skyline^®^ software (https://skyline.ms/project/home/begin.view). The verification of uniqueness of peptides was realized with BLAST^®^ software (http://blast.ncbi.nlm.nih.gov/Blast.cgi).

Then, a trypsin digestion from a pure solution of PBZ was experimentally performed. Among the signature peptides determined *in silico*, only the peptides contained in SIL-PBZ-like and showing the highest abundance in Full Scan and PRM mode were selected. Finally, ten blank plasmas prepared as described below were analyzed in order to confirm the selectivity of the selected surrogate peptides.

#### 2.1.4. Sample Preparation

**Figure 1:**
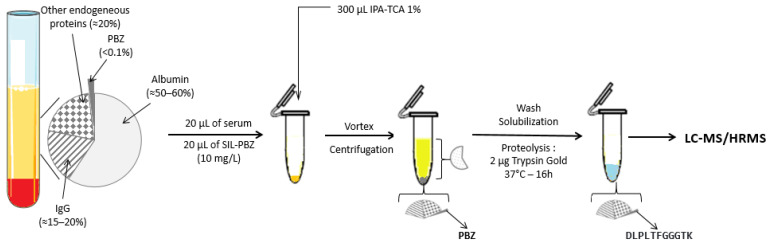
Sample preparation protocol. PBZ: pembrolizumab; IgG: immunoglobulin G; DLPLTFGGGTK: LC8 surrogate peptide; IPA-TCA 1%: isopropanol and 1% of trichloroacetic acid.

The sample clean-up strategy consisted of a selective precipitation of proteins to deplete only albumin, which consisted of about 60% of plasma proteins (Figure 1).

An aliquot of 20 µL of SIL-PBZ-like at 10 µg/mL was added to 20 µL of plasma. Then, 400 µL of isopropanol containing 1% of trichloroactetic acid was added in a low adsorption Eppendorf. The extraction tubes were vigorously mixed and centrifuged at 1500 g for 5 min. The supernatant including albumin was removed. Then, the pellet was resolubilized in 200 µL of methanol to both wash the pellet and remove acid, before a second quick centrifugation at 2000 rpm for 2 min. After removing the supernatant, the pellet was re-suspended in 45 µL of ammonium bicarbonate (100 mM). A quantity of 2 µg of Trypsin Gold (5 µL at 0.4 µg/µL) was added to perform proteolysis, and eppendorfs were stored at 37 °C overnight. After centrifugation (13,000× *g*, 5 min), 20 µL of clear supernatant was injected into the chromatographic system.

#### 2.1.5. Method Validation

Selectivity, linearity, accuracy, precision, recovery, carryover and stability were tested for the method validation and acceptance criteria were defined as recommended by the European Medicines Agency (EMA) Guidelines [18].

##### Selectivity

To evaluate the selectivity of our method, 10 double blank human plasmas (neither PBZ nor SIL-PBZ-like) were prepared as described before being analyzed. Then, in order to ensure the lack of interference from other mAbs, blank plasmas were spiked with nivolumab, ipilimumab, cetuximab or rituximab and were analyzed. Selectivity was confirmed when the area of peak of interest was less than 20% of the lower limit of quantification (LLOQ) and 5% of SIL-PBZ-like.

##### Linearity, Accuracy and Precision

A six-point standard calibration curve was prepared by spiking blank plasmas in range of 1 µg/mL to 100 µg/mL (1, 2.5, 7.5, 20, 50, and 100 µg/mL). Quantitative data were obtained using an area ratio between PBZ and SIL-PBZ-like. Evaluation of linearity was performed on seven different days using the squared correlation coefficient (R²). Linearity was confirmed when CV% and bias% were <±15% (except LLOQ).

Four internal quality control (IQC) levels were prepared at 1 (LLOQ), 5, 25, and 80 µg/mL to evaluate analytic performances of our method. Precision (intra- and inter-day) and accuracy were assessed by assaying six replicates (four replicates for LLOQ) of each level on four different days. The acceptability criteria were: precision, defined as the coefficient of variation (CV%), should be less than 15% (20% for LLOQ) and satisfactory accuracy with recovery between 85% and 115% (bias < ±20% for LLOQ). Inter-day precision was also evaluated on seven plasma samples of treated patients on five different days.

##### Recovery and Matrix Effect

Total recovery (TR %) of PBZ was determined by analyzing low IQCs and high IQCs in triplicate. It was assessed by the comparison of signals of PBZ obtained from extracted plasma samples and samples spiked directly in a solution of ammonium bicarbonate (Equation (1)). The matrix effect (ME %) was also evaluated on the same samples, comparing peak responses of SIL-PBZ-like (20 µg/mL) added in the ammonium bicarbonate and in post-extracted samples (Equation (2)). Extraction yield (ER %) was calculated by dividing total recovery and the matrix effect (Equation (3)). Results were expressed as a ratio of responses as in the equations below:

(1) $$\mathrm{TR}\;\%=\frac{{\mathrm{PBZ}}_{\mathrm{plasma}}}{{\mathrm{PBZ}}_{\left({\mathrm{NH}}_{4}{\mathrm{HCO}}_{3}\right)}}$$

(2) $$\mathrm{ME}\;\%=\frac{\mathrm{SIL}-\mathrm{PBZ}-{\mathrm{like}}_{\mathrm{plasma}}}{\mathrm{SIL}-\mathrm{PBZ}-{\mathrm{like}}_{\left({\mathrm{NH}}_{4}{\mathrm{HCO}}_{3}\right)}}$$

(3) $$\;\mathrm{ER}\;\%=\frac{\mathrm{TR}}{\mathrm{ME}}$$

##### Stability

Freeze/thaw stability (three cycles at room temperature to −20 °C) and long-term stability (at −20 °C) were simultaneously determined by re-analyzing patient samples on different days. Post-extraction stability was determined by re-analyzing calibrators and IQCs after leaving them at room temperature for 48 h.

##### Carry-Over

Carry-over was evaluated by analyzing double blank plasma samples right after injection of the upper limit of quantification (ULOQ) sample. An acceptable carry-over was a surface with less than 20% of the mean area of LLOQ.

### 2.2. ELISA

The method was adapted and slightly modified from a previous home-made method for the quantification of nivolumab in human plasma [19]. Then, the method was validated in accordance with the EMA recommendations (calibration curve, precision, and accuracy within-run and between-run, specificity, selectivity, and dilution integrity) [18].

Briefly, 100 µL of pre-diluted sample (1:12,000 in blocking buffer) was contacted for 2 h with human recombinant PD1/Fc pre-coated in a 96-well-plate. After a washing step, alkaline-phosphatase (ALP) conjugated mouse anti-human IgG4-Fc was added as a secondary antibody. This mixture remained incubated at +4 °C overnight. Then, after another washing step with PBS containing 0.05% Tween 20, p-nitrophenylphosphate was added to react with ALP for 30 min in the dark at room temperature. After stopping the reaction with 50 µL of NaOH (3 N), determination of the concentration of PBZ was assessed quantifying the yellow product of the reaction by measuring the absorbance at 405 nm.

The calibration curve was constructed with six calibrators (2.5, 5, 10, 25, 37.5, and 50 µg/mL). Samples with PBZ levels above 50 µg/mL were re-analyzed after a 1:2 dilution in blank plasma.

### 2.3. Application

During a regular medical visit, blood samples (5 mL) from cancer patients treated with PBZ for NSCLC or melanoma were collected at steady state in heparin lithium-containing tubes just before the next drug intake (trough concentration). The samples were centrifuged (1850× *g*, +4 °C, 10 min), and then the plasma was collected and transferred into propylene test tubes before storage at −20 °C up to subsequent analysis. The local Review Board for Oncology approved this study. Overall, 70 plasma samples from cancer patients could be analyzed with the LC-MS/HRMS and the in-house ELISA methods. The results were then compared using Passing-Bablok [20] regression analysis and Bland-Altman plotting [21].

## 3. Results

### 3.1. Selection of Proteotypic Peptides and Selectivity with LC-MS/HRMS

**Figure 2:**
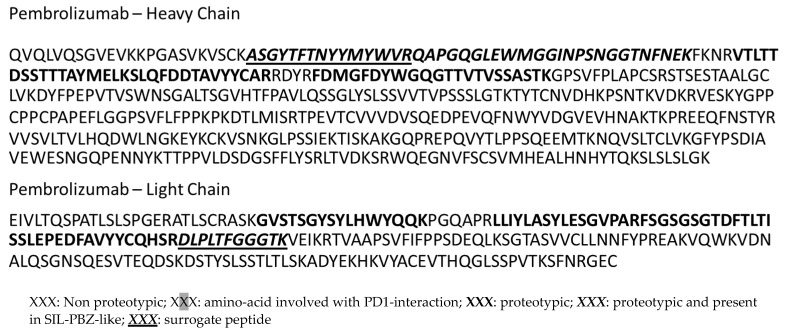
Amino-acid sequences of the heavy chain (HC) and the light chain (LC) of pembrolizumab (PBZ).

Humanized monoclonal antibodies, such as PBZ, presents more proteotypic peptides than fully human mAbs like nivolumab. Proteotypic peptides are usually localized on the complementary determining region (CDR), which contains amino acids interacting with their target, as PD1 for PBZ [22,23]. As described by Lee et al., the paratope of PBZ consists of 15 AA on the heavy chain and 13 AA on the light chain (Figure 2) [22]. LC8 (light chain, peptide number 8) and HC5 (heavy chain, peptide number 5) peptides contain some of those amino-acids involved in the interaction with PD-1.

**Figure 3:**
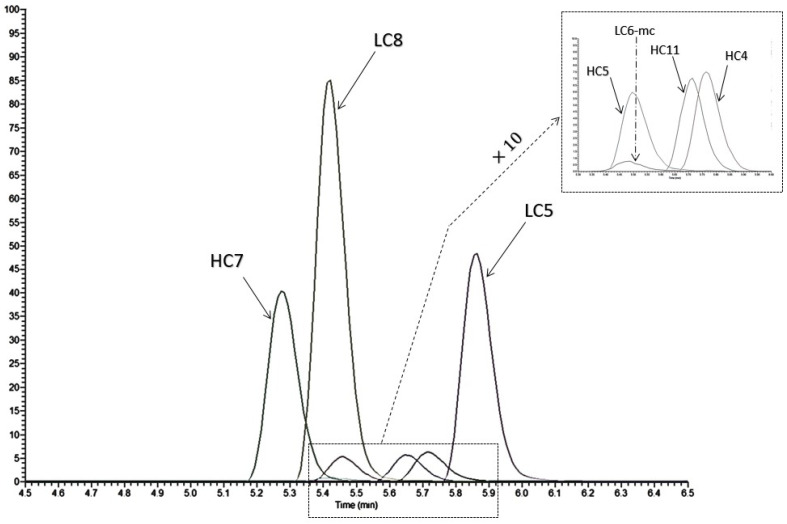
Chromatogram of proteotypic peptides obtained in Full Scan mode analysis. Sample was from a pure solution of pembrolizumab (20 µg/mL) after proteolysis by trypsin.

In silico data indicated that PBZ presented nine tryptic proteotypic peptides. Among them, only two peptides, DLPLTFGGGTK (LC8) and ASGYTFTNYYMYWVR (HC5), were also present in SIL-PBZ-like and were detectable in Full Scan mode in plasma (Figure 3).

The first tests at 1 µg/mL in matrix (LLOQ fixed) showed that only LC8^2+^ (*m*/*z* = 553.2980) was sensitive enough for satisfactory detectability and quantification. Thus, the quantification of PBZ was only based on this surrogate peptide (Table 1).

**Figure 4:**
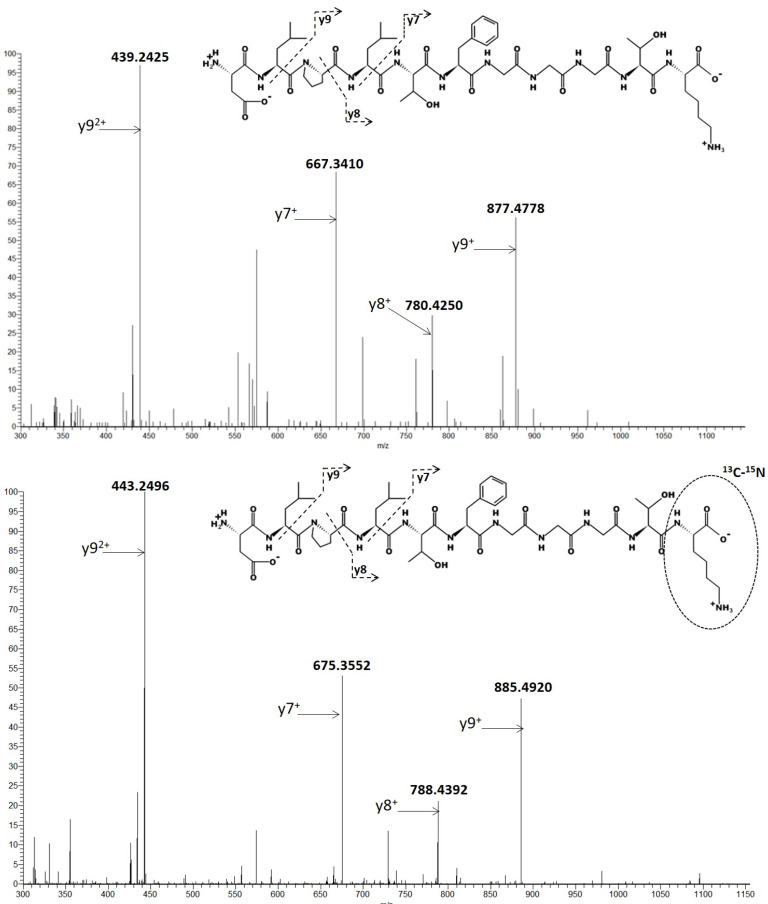
MSD2 spectra of PBZ (up) and SIL-PBZ-like (down) of a G1 sample (1 µg/mL and 10 µg/mL respectively). Peptide sequence of DLPLTFGGGTK (LC8) and daughter ions are represented on the bottom right of each figure. The +2 forms of LC8 were selected as precursor ions with *m*/*z* = 553.2980 and 557.3051 for PBZ and SIL-PBZ-like, respectively.

According to their intensities, fragmented ions y9^2+^ and y7^+^ were summed for the quantification of the surrogate LC8 peptide (Figure 4).

**Figure 5:**
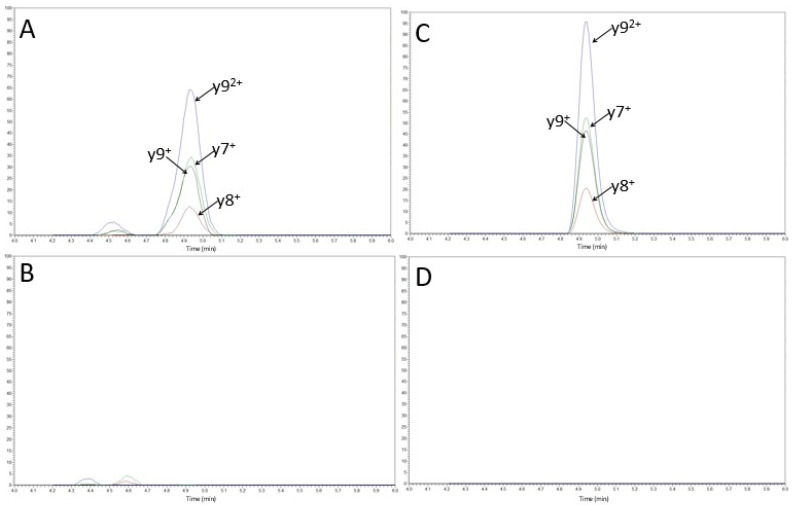
Chromatograms of a standard G1 (1 µg/mL of PBZ (**A**) and 10 µg/mL of SIL-PBZ-like (**C**)) and a double blank sample (**B**,**D**).

Analysis of 10 double blank plasma samples (no PBZ or SIL-PBZ-like added) did not show any interference (Figure 5). Likewise, no signal was observed from samples spiked with nivolumab, ipilimumab, rituximab, or cetuximab.

### 3.2. Validation with LC-MS/HRMS Method

#### 3.2.1. Linearity, Accuracy, Precision and LLOQ

Quantification was performed using a weighted (1/X) quadratic regression curve generated by plotting the peak area ratio of the most abundant daughter ions from LC8^2+^ (y9^2+^ + y7^+^) to SIL-PBZ-like versus PBZ concentration (µg/mL). The quadratic model was selected based on the use of Mandel’s test to check for nonlinearity [24]. As already described for the analysis of mAbs by mass spectrometry, summing the signals of y9^2+^ and y7^+^ allowed for both increasing the signal and overcoming variations in fragmentations.

Linearity was evaluated on seven different calibration curves (Table 2) with acceptable performances (CV% < 15%, bias < 15%, R² > 0.99).

Accuracy and precision of IQC samples are reported in Table 3 and reproducibility on patient samples in Table 4. The LLOQ was set at 1 µg/mL, whereas within- and between-day precision were below 15% for different IQC levels. These performances met all the recommendations for the validation of the assay methods proposed by EMA [18].

#### 3.2.2. Recovery and Matrix Effects

Total recovery, which corresponds to sample preparation recovery (including proteolysis yield), and the matrix effect were calculated at two different concentrations. The matrix effect was calculated with the SIL-PBZ-like. Extraction recoveries were calculated at 75.3 ± 9.9% for LQC and 62.7 ± 10.9% for HQC, and matrix effects were determined at 39.6 ± 11.2% and 36.7 ± 10.5% for LQC and HQC, respectively. Thus, absolute recoveries were calculated at 29.6 ± 5.6% for LQC and 23.0 ± 4.6% for HQC. These results confirm the predominant role of a suitable internal standard to correct all the variations.

#### 3.2.3. Carryover and Sample Stability

Analysis of the double-blank samples immediately after the injection of the highest calibrator did not show any carryover.

Pre-analytical stability tests did not show any degradation, either at −20 °C or after three freeze-thaw cycles from patient samples (Table 4). In fact, the concentrations measured after four months at −20 °C and after three freezing-thawing cycles had an average bias of −7% compared with the initial measurement. In addition, the post-analytical stability study of PBZs, with reinjection of calibrators and IQCs after 48 h at +4 °C, did not show any particular degradation (<12%).

### 3.3. Validation with ELISA Assay

**Figure 6:**
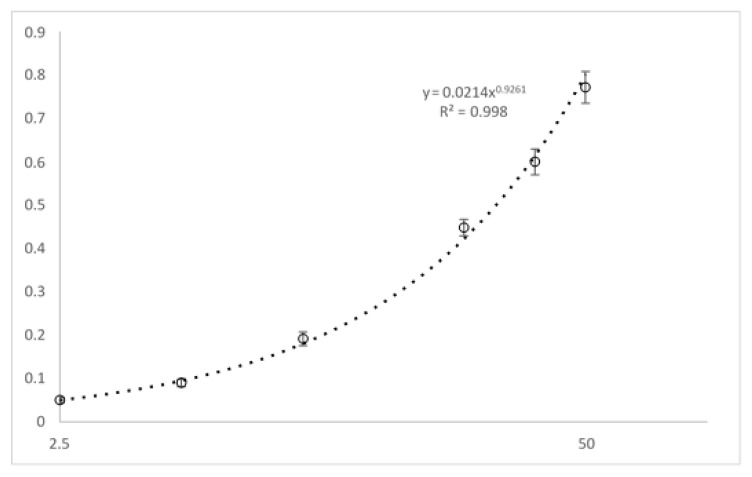
Analysis of 70 patient samples with LC-MS/HRMS and ELISA. Passing–Bablok regression (left) and Bland-Altman (right) analysis between the LC-MS/HRMS method and the ELISA method. For Passing–Bablok analysis, the solid and dashed lines indicate the regression line and confidence interval for the regression line, respectively. For Bland-Altman analysis, the solid line indicates the mean difference between the methods.

The calibration range for PBZ was from 2.5 to 50 µg/mL. The signal (optical density, OD) according to PBZ concentration was fitted by using a power regression equation (y = 0.0214x^0.9261^) (Figure 6). Coefficients of variation for within and between run were less than 9.9% for the quality controls (5, 20, and 40 µg/mL) and less than 14.1% for the LLOQ (2.5 µg/mL). Within-day and between-day accuracies were ranged from 92.4 to 105.7%. No analytical interference was detected in blank samples (*n* = 6).

### 3.4. Comparison of Methods Based on Samples from Treated Patients

The quantification of PBZ on 70 plasma samples from treated patients showed that 68 results were within the dynamic range of the calibration curve of LC-MS/HRMS. Only two samples had a PBZ concentration below the LLOQ for LC-MS/HRMS (1 µg/mL) and ELISA (2.5 µg/mL), respectively.

**Figure 7:**
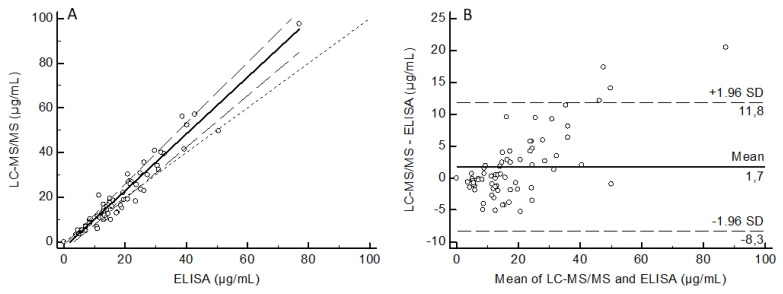
Analysis of 70 patient samples with LC-MS/HRMS and ELISA. Passing-Bablok regression (**A**) and Bland-Altman (**B**) analysis between the LC-MS/HRMS method and the ELISA method. For the Passing-Bablok analysis, the solid and dashed lines indicate the regression line and confidence interval for the regression line, respectively. For the Bland-Altman analysis, the solid line indicates the mean difference between the methods.

Agreement between LC-MS/HRMS and ELISA results was assessed by Passing-Bablok regression and Bland-Altman plot (Figure 7). Regression analysis showed that these two methods were correlated (Pearson r² = 0.94, *p* < 0.001, *n* = 70). The regression equation of the Passing-Bablok analysis was LC-MS/HRMS= 1.27 (95% CI: 1.15 − 1.36) × ELISA- 2.57 (95% CI: −3.64–−1.38). Bland-Altman analysis did not show a significant difference between the two methods, with a mean bias of 1.7 (95% CI: −8.3–11.8).

## 4. Discussion

As is usually done to quantify monoclonal antibody with mass spectrometry, we developed a bottom-up method to quantify pembrolizumab. LC8 was selected as the surrogate peptide, especially because it was the most abundant among the candidate peptides. Then two ions (y9^2+^ and y7^+^) were used for the quantification and two other ions (y9^+^ and y8^+^) were used as qualifiers ions. In the previous method, based on quadripolar tandem mass spectrometer, LC8 was also selected as a surrogate peptide but only the transition *m*/*z* 553.4 → 667.4 (corresponding to y7^+^) was used. Chiu et al. used two I.S.: the first one (tocilizumab, humanized IgG1) added in each sample at the beginning of the sample preparation and was used to correct pre-analytical variations (SPE recovery, proteolysis recovery) [16]. The second I.S. (a peptide with a 13C-15N-Valine labelled) was post-column infused during the chromatographic analysis to correct the matrix effect. In the present work, SIL-PBZ-like (IgG4) containing arginine and lysine labelled with stable isotope appeared as an easier way to correctly quantify PBZ. This I.S. corrects every variation (extraction, proteolysis, matrix effect) and assures reliable performances to quantify PBZ.

Albumin depletion was selected as sample preparation for different reasons. It was cheaper, less time-consuming, and easier to perform in routine practice than other protocols, such as IgG immunocapture as used by Chiu et al. for PBZ quantification [16]. Albumin depletion allows for the cleaning-up of samples by removing more than 50% of endogenous proteins. In the case of PBZ, the excellent analytical response of LC8 and the selectivity of HRMS allowed for reaching a LLOQ at 1 µg/mL. However, for other mAbs it may be necessary to implement a more selective sample preparation [25,26].

Few methods of quantification of PBZ have been already described (Table 5).

Numerous clinical studies have studied different treatment regimens and different doses for the administration of PBZ: dose based on body weight (1 to 10 mg/kg) or flat dose (200 or 400 mg), and administration every two, three, or six weeks according to the dose [6]. However, recent studies suggest a new approach based on a reduction in the doses administered every 3 weeks. This strategy would achieve the same therapeutic efficacy while significantly reducing the cost of treatment [11]. Thus, plasma monitoring of PBZ could be helpful in cancer patients to ensure therapeutic drug exposure following alternative dosing schedules. Previous pharmacokinetic studies have reported peak (Cmax) and trough (Cmin) concentrations at a steady state around 90 and 30 µg/mL, respectively, at 200 mg every 3 weeks [6,27]. Cmin are equivalent between 200 mg every 3 weeks and 400 mg every 6 weeks. After a single infusion, one study found a mean trough PBZ concentration at 12.7 µg/mL (9.1–15.1 µg/mL, *n* = 8) [12]. The two published ELISA methods indicated the same range of concentrations while a recent assay based on LC-MS/MS was from 5 to 800 µg/mL [12,13,16]. In the present study, the dynamic range of standard was from 1 to 100 µg/mL. The plasma concentrations assayed in 70 samples from cancer patients with PBZ ranged from 3.3 to 97.5 µg/mL.

This result suggests that the present method is suitable for quantification of PBZ with current dosing regimens as well as for potential future low-dose administration.

## 5. Conclusions

We developed and validated a selective, precise, and accurate LC-MS/HRMS for PBZ quantification in plasma from cancer patients. This method was based on the use of a simple sample preparation procedure and a stable-isotope-labeled PBZ-like internal standard. It was successfully applied in 70 plasma samples from cancer patients and cross validated with the ELISA method. Overall, the present LC-MS/HRMS method is suitable for the plasma quantification of PBZ in the context of PK/PD studies or TDM.

## Figures and Tables

**Table 1:** LC8 surrogate peptide used for pembrolizumab (PBZ) quantification by LC- MS/HRMS method using the internal standard (SIL-PBZ-like).

Compound.	Selected Peptide	Precursor Ion		Product Ion		
		(*m*/*z*)	Charge	Ion	(*m*/*z*)	Charge
PBZ	DLPLTFGGGTK	553.2980	+2	y9 y7 y9 y8	439.2425 667.3410 877.4778 780.4250	+2 +1 +1 +1
SIL-PBZ-like (I.S.)	DLPLTFGGGTK	557.3051	+2	y9 y7 y9 y8	443.2496 675.3552 885.4920 788.4392	+2 +1 +1 +1

I.S.: Internal Standard (full-length stable isotope-labeled pembrolizumab-like); Amino acid in bold was ^13^C-^15^N-Arginine.

**Table 2:** Inter-day validation (*n* = 5 days) for the determination of pembrolizumab in plasma (*n* = 7).

	Quantifying ions (y9^2+^ + y7^+^)		
Spiked (µg/mL)	Found (µg/mL) (mean ± s.d.)	Precision (%)	Accuracy (%)
1	1.0 ± 0.1	9.6	95.4
2.5	2.7 ± 0.2	7.6	108.6
7.5	7.6 ± 0.9	12.3	98.2
20	21.3 ± 1.4	6.5	106.3
50	47.9 ± 2.3	4.9	95.7
100	101.0 ± 1.2	1.2	101.0

s.d.: standard deviation.

**Table 3:** Assessment of accuracy and precision. Data were from six replicates (5, 25, and 80 µg/mL) and four replicates (1 µg/mL) and analyzed on four different days.

Concentration		Precision (%)		Accuracy (%)	
Spiked	Found (mean ± s.d.)	Within-Day	Between-Day	Within-Day	Between-Day
1 (LLOQ)	1.0 ± 0.2	9.8	17.6	89.7	102.2
5	4.9 ± 0.6	6.3	11.4	102.8	97.2
25	23.9 ± 3.8	11	12.2	103.7	95.6
80	84.1 ± 12.8	7.8	14.1	101.4	105.1

s.d.: standard deviation.

**Table 4:** Reproducibility and long-term stability (−20 °C over four months, with three freeze-thaw cycles) using sample re-analysis of seven patient samples.

Patient	Found (µg/mL) (mean ± s.d.)	Reproducibility (%)	Difference (%)
P1 P2 P3 P4 P5 P7 P9	4.6 ± 0.7 4.3 ± 0.7 11.1 ± 1.4 <1 10.9 ± 1.3 34.5 ± 5.7 43.2 ± 4.9	14.2 15.8 12.6 - 11.5 16.5 11.3	−4% −12% −11% - −3% −2% −9%

s.d.: standard deviation.

**Table 5:** Main characteristics of the assays published for pembrolizumab quantification and the present study.

Study	Method	Standard Curve and LLOQ	Precision
Basak et al. [12]	ELISA	0.80–100 µg/mL	Data not available
Pluim et al. [13]	ELISA	2–100 µg/mL (LLOQ 2 µg/mL)	CV < 6.6% CV < 5.8%
Chiu et al. [16]	LC and triple quadrupolar mass spectrometer Two internal standards (tocilizumab and post-infused labelled peptide)	5–800 µg/mL (LLOQ at 3 µg/mL)	CV < 7.1% Data not available
Millet et al. (Present study)	LC and tandem quadrupolar and high resolution (Orbitrap) mass spectrometer SIL-PBZ-like as internal standard ELISA	1–100 µg/mL (LLOQ at 1 µg/mL) 2.5–50 µg/mL (LLOQ at 2.5 µg/mL)	CV < 14.1% CV < 17.6% CV < 9.9% CV < 14.1%

LC: liquid chromatography, LLOQ: lower limit of quantification. The between-days precision is mentioned in the table.

## Data Availability

We can provide data on demand.
